# Topologically Organized Networks in the Claustrum Reflect Functional Modularization

**DOI:** 10.3389/fnana.2022.901807

**Published:** 2022-06-23

**Authors:** Gao Xiang Ham, George J. Augustine

**Affiliations:** Lee Kong Chian School of Medicine, Nanyang Technological University, Singapore, Singapore

**Keywords:** claustrum, networks, connectivity, functional modules, claustrum topology, connectome

## Abstract

Using genetic strategies and viral-based directional tracers, we investigated the topological location and output networks of claustrum (CLA) neuron populations projecting to either the retrosplenial cortex, primary motor cortex, or basolateral amygdala. We found that all three CLA neuron populations clearly reside in distinct topological locations within the CLA complex and project broadly to multiple downstream targets. Each neuron population projects to different targets, suggesting that each CLA subzone coordinates a unique set of brain-wide functions. Our findings establish that the claustrum complex encompasses at least three minimally overlapping networks that are compartmentalized into different topological subzones. Such modularity is likely to be important for CLA function.

## Introduction

Almost all hypotheses regarding the function of the claustrum (CLA) are based on the extensive brain-wide connectivity of this poorly-understood brain region (Crick and Koch, 2005; Smythies et al., 2012; Mathur, 2014; Goll et al., 2015; Yin et al., 2016; Dillingham et al., 2017; Smith et al., 2019). However, investigation of the claustral connectome has led to divergent views regarding the functional organization of the CLA. Early neuroanatomical studies on cats and primates found that injection of retrograde tracers into different cortical regions labelled CLA neurons that were clustered into projection-based subzones with varying degrees of overlap (Macchi et al., 1983; Sherk, 1986; Gattass et al., 2014). Such results indicate that the CLA is divided into topological regions with distinct functions (Baizer et al., 2014). Consistent with this notion, receptive field maps associated with vision and audition map onto completely distinct claustrum regions (Olson and Graybiel, 1980; Remedios et al., 2010).

However, replication of these tracing experiments in rodents has yielded conflicting results. Rather than the distinct modularization described in cats and primates, numerous studies indicate that neurons in the rodent CLA project to multiple cortical targets (Kitanishi and Matsuo, 2017; Zingg et al., 2018; Peng et al., 2021), often indicated by co-labeling of CLA neurons following retrograde tracer injection into pairs of cortical regions. 3D reconstruction of single CLA neurons also reveals extensive axon networks that appear to innervate both brain hemispheres and—in extreme cases—even wrap around the entire brain (Peng et al., 2021). These findings are at odds with the concept of projection-based CLA topology. Instead, the multi-region output from individual CLA neurons suggests an ability to broadcast information widely across the brain. These findings rekindle hypotheses that the claustrum serves as a global integrator, performing holistic functions such as generation of consciousness (Crick and Koch, 2005) or multisensory perceptual integration (Smythies et al., 2012).

Although these multi-projecting properties could be unique to the less-evolved CLA of rodents, recent studies have also uncovered evidence of a modular rodent CLA. Chia et al. (2020) showed that CLA neurons projecting to different brain regions preferentially receive synaptic input from functionally related cortices. Marriott et al. (2021) also demonstrated—using retrograde tracing from four regions simultaneously—that CLA neurons are apparently organized topologically, according to their downstream projection targets. Marriott et al. (2021) observed a gradient-like distribution of CLA neurons that maps anterior-posterior cortical targets onto the dorsal-ventral axis of the CLA. They also observed that up to 30% of CLA neurons project to multiple targets, suggesting that multiple cortical targets do receive input from the same CLA neurons. Together, these studies suggest that functional modularization may exist in the rodent CLA, even in the presence of brain-wide axonal arborization.

To reconcile these somewhat divergent views of CLA organization, we hypothesize that topologically-distinct populations of CLA neurons may also project to *distinct* downstream networks; for example, one CLA neuronal population could project to sensorimotor cortices, while another CLA neuronal population could project to a different network, such as association cortices. We tested this hypothesis in three ways. First, using a retrograde Cre approach, we determined the topological organization of CLA neuron populations projecting to three different initial targets: the primary motor cortex (M1), retrosplenial cortex (RSC), and the basolateral amygdala (BLA). Next, we identified co-projection targets of CLA neurons projecting to these three initial targets and, finally, we compared the degree of overlap between their projection networks. We found that neurons projecting to M1, RSC and BLA reside in distinct topological zones within the CLA and participate in vastly different projection networks. These distinct projection networks reveal that the CLA can be divided into a minimum of three topologically-distinct functional modules that may provide concerted influence on different sets of brain regions. The existence of distinct functional networks within the CLA complex reveals how the rodent CLA is organized and will guide future experiments that determine whether these specific CLA neuron populations subserve different brain functions.

## Materials and Methods

### Animals

A total of 11 C56/BL6J wild-type male mice were used for the experiments: three mice for M1 and RSC initial targets, and five mice for experiments where the BLA was the initial target. Mice were 2–3 months old at the time of stereotaxic surgery for tracing experiments. All animal procedures were performed according to the guidelines of the NTU Institutional Animal Care and Use Committee.

### General Surgical Procedures for Viral Expression of Target Genes

Mice were deeply anesthetized using isoflurane and placed in a stereotaxic frame equipped with a heating pad. Animals were monitored for breathing and their body temperature was checked throughout the surgery. For pre-surgery analgesic relief, buprenorphine and lidocaine were injected subcutaneously above the back and incision site, respectively. A skin incision was made, followed by craniotomy and dura removal above the target coordinates. Precise viral injections were made using glass pipettes with tip diameters of 30–50 μm and a pressure injector (UltraMicroPump3 and SMARTouch Controller; World Precision Instruments, Sarasota, FL). Both the glass pipettes and Hamilton syringe (5 μl model with RN Compression Fitting; Reno, NV) were first filled with mineral oil before front filling with viruses. Viruses were injected at 1–2 nl/s into each target region. Coordinates are defined based on the following convention: Medial-lateral (ML), anterior-posterior (AP), depth based on dura (DV) and angle represents deflection from midline with pipette tip pointing toward the center where applicable. The coordinates used for different brain regions are as follows: RSC (ML: 0.5, AP: −2.5, DV: 0.8, Angle: 8°), M1 (ML: −1.8, AP: 1.7, DV: −0.8), BLA (ML: −3, AP: −1.55, DV: −4.6), CLA (ML: −2.7, AP: 1.3, DV: −2.9). Injection pressure was allowed to equalize for 5 min before retraction of the glass pipette, followed by stitching up of the incision and recovery on a warming pad. Mice were monitored and supplied with Meloxicam and Baytril in their drinking water for 1-week post-surgery.

### Expression of Fluorescent Proteins in Projection-Defined CLA Neurons

**Figure 1:**
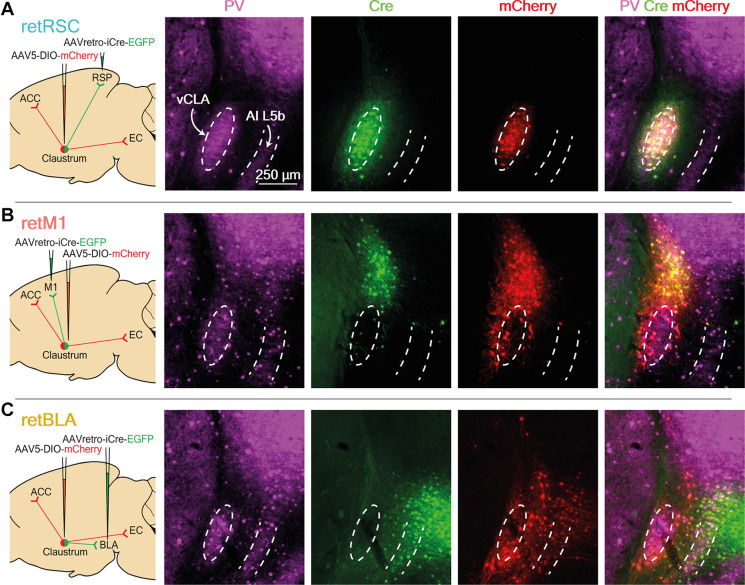
Neurons projecting to RSC, BLA, and M1 reside in distinct topological zones within the claustrum (CLA). **(A)** Images of RSC-projecting CLA neurons (retRSC), **(B)** BLA-projecting CLA neurons (retBLA), and **(C)** M1-projecting CLA neurons (retM1). (Left column) Viral strategy to target projection-defined claustrum neurons and map output networks. (2nd-4th columns) Parvalbumin (PV) staining and resultant viral expression of Cre and mCherry. Dotted white ellipses demarcate the ventral CLA core (vCLA) while dotted white lines demarcate layer 5b of the agranular insula, (AI L5b) as indicated by PV immunohistochemistry. Cre expression produced by retrograde AAV injected into the initial targets (RSC, M1, and BLA), as indicated by EGFP-labelled neurons that project to the initial targets. Cre-dependent mCherry expression in CLA neurons used for network mapping. (Right column) Merged images of all three fluorescence channels.

To map the axonal networks of projection-defined CLA neurons, a Cre-Lox system was implemented *via* two AAV injections per animal (see Figure 1 below). Retrograde AAVs expressing iCre and EGFP (AAVretro-hSyn-EGFP-iCre—VVF-ZNZ; 100 nl; 10E12 viral genomes/ml) were injected into one of three initial targets, M1, RSC or BLA. A conditional AAV expressing Cre-dependent mCherry (AAV5-EF1a-DIO-mCherry, from the UNC vector core; 150 nl; 10E12 viral genomes/ml) was subsequently injected into the CLA to express mCherry in CLA neurons projecting to the initial targets.

Because the insula projects strongly to the BLA, additional precautions were taken when the BLA was the initial target. In these experiments, Cre was also expressed in many deep layer insula neurons that are located close to the CLA (see Figure 1C, Cre below). Therefore, the injection coordinates for the Cre-dependent viral vector were adjusted (300 μm shift toward midline @ ML −2.4) to deliberately position viral spread away from the insula, while encompassing the CLA. This allowed selective expression of mCherry only in BLA-projecting CLA neurons and not in insula neurons. Any animals exhibiting strong somatic expression of mCherry in the insula were excluded from our analysis (*n* = 2).

### Histology and Immunohistochemistry

To allow adequate time for fluorophore expression, animals were perfused and sacrificed 2–4 weeks post-surgery. Transcardial perfusion was performed using saline, followed by 4% paraformaldehyde (PFA) in phosphate buffered saline (PBS). Dissected brains were post-fixed in 4% PFA overnight, followed by 30% sucrose solution in PBS for 2 days, or until brains sank. Brain sections (60 μm thickness) were first washed, blocked and stained with rabbit-anti-PV (1:1,000, Swant: PV-28, RRID:AB_2315235), chicken-anti-GFP (1:1,000, Abcam:AB_13970, RRID:AB_300798) and rat-anti-mcherry (1:1,000, mCherry Monoclonal Antibody (16D7) from Thermo Fisher Scientific, RRID:AB_2536611) primary antibodies overnight at RT, or for 2 days at 4°C. These sections were then washed and treated with secondary antibodies (1:1,000, Alexa Fluor 488, Alexa Fluor 568, and Alexa Fluor 647; RRID:AB_142924, RRID:AB_2534121, RRID:AB_2536101) at RT for 2–3 h. Stained sections were also stained with DAPI, to visualize cell nuclei.

### Image Acquisition and Preprocessing

Images were acquired with a ZEISS Axio Scan.Z1 slide scanner. To reduce the out-of-focus blur associated with such wide-field imaging, stained sections were imaged at different focal depths (using z-stack) in the EDF mode (Extended Depth of Focus), which combines sharp images from each z-slice to reconstruct a flat projection. Tiled images were subsequently flat-field corrected and stitched using the WholeBrain program (Fürth et al., 2018). All images were processed using custom R scripts and ImageJ macros to rotate slices and separately process the different fluorescent channels. Anterograde-labeled axons were extracted using a Hessian filter—FeatureJ^1^; “Largest eigenvalue of Hessian tensor” and “Smoothing scale” factor set to 1 (Grider et al., 2006), which enhances the features of curvilinear fibers, followed by inversion and background subtraction (sliding paraboloid, 50 pixel radius, enable smoothing) to enhance image contrast.

### Injection Site Quality Control

Injection sites were inspected in each animal to confirm that viruses were injected into the target regions. Animals in which fluorescence was not localized to the target regions were excluded from the analysis.

### Axon Network Quantification

Quantification of axon networks was performed using processes adapted from the QUINT workflow (Yates et al., 2019). The QUINT workflow contains several standalone programs and was chosen because it facilitates both the re-slicing of atlas planes to correct for deviations in sectioning angle and the warping of brain regions to account for mounting shear. These processing steps ensure proper delineation of brain regions and the correct assignment of axon labeling.

All sections were first manually registered to the Allen mouse brain atlas (Wang et al., 2020) using the PV channel. Differences in PV expression across layers and brain regions allowed objective alignment of brain sections. Registration was done using QuickNII (Puchades et al., 2019) which allows virtual re-slicing of atlas sections to match actual slice angles. VisuAlign, a tool packaged with QuickNII, was used to warp brain regions to account for the shearing of regions during mounting.

To quantify the density of axon innervation throughout the brain, the contrast-enhanced axon images were binarized *via* auto-thresholding and subsequently quantified using Nutil (Groeneboom et al., 2020) to generate axon densities for each brain region segmented according to the PV-registered atlas maps. Axon densities were calculated as the number of axon-labeled pixels divided by the number of pixels in each region.

Due to the cytoplasmic nature of mCherry labeling and the proximity of Layer 6b (L6b) to the anterior commissure, axons traveling through the anterior commissure were often quantified as axon densities in L6b of various cortices. The narrowness of L6b fibers in the atlas could combine with slight deviations in atlas registration to yield substantial misrepresentation of axon densities for L6b of many cortices. Hence the axon densities assigned to L6b of all cortical regions were excluded from our analysis. In addition, regions were deemed to be innervated only if axonal density was more than a threshold (0.002 pixels/area) that was empirically determined based on the distribution of axonal fluorescence. Threshold-identified regions were additionally verified for axonal expression of mCherry in the raw images. To account for variability in mCherry expression between animals and variability in absolute fluorescence intensity across animals, axon density from each brain region (axon pixels/pixels in brain region) was quantified as a fraction of the total axonal density across all innervated regions.

### Statistical Analysis

Statistical comparisons were performed using custom R scripts. To compare differences in the axon network of the three experimental conditions (*n* = 3 mice for each condition), an ANOVA test was performed per region, using the axon density from each experiment to compare innervation across the three experimental conditions. *Post-hoc* Tukey’s HSD was performed following ANOVA to obtain significance values for comparisons across experimental pairs for each brain region.

## Results

### CLA Neurons Projecting to RSC, M1, and BLA Reside in Distinct Topological Zones

To determine whether different projection networks exist within the CLA, we investigated the output networks of CLA neurons that project to three different initial targets: the retrosplenial cortex (RSC), the basolateral amygdala (BLA), and the primary motor cortex (M1). Among the many regions innervated by the CLA, these were selected to reflect different cortical hierarchies: higher/association cortex (RSC), sub-cortex (BLA), and sensorimotor cortex (M1). A viral strategy, based on Cre-lox, allowed us to visualize the axon arborizations of projection-defined CLA neurons by restricting expression of mCherry to CLA neurons that project to one of the three initial targets (Figure 1, left-most column). Retrograde adeno-associated virus (retro-AAV) packaging EGFP and Cre recombinase were first injected into one of the three initial targets, followed by injection of a conditional AAV (AAV5) carrying a Cre-dependent mCherry vector into the vicinity of the CLA. *Post-hoc* parvalbumin (PV) immunohistochemistry was used to define the ventral (vCLA) core and layer 5b of the agranular insula (AI; Figure 1, PV).

To confirm the specificity of our targeting strategy and the topology of CLA neurons projecting to different targets, we examined the expression of Cre and mCherry in the CLA (Figure 1, Cre and mCherry columns). Because the exact boundaries of the CLA are a topic of debate, we have adopted the more inclusive CLA complex definition of Smith et al. (2018), (2020). Strong expression of Cre in the CLA was observed following virus injection into either the RSC or M1 initial targets (Figures 1A,B, Cre). RSC-projecting neurons were confined within the vCLA core, while M1-projecting neurons clustered in a zone above the vCLA core that is sometimes called the dorsal CLA (dCLA; Smith et al., 2018, 2020; Zingg et al., 2018). These strong and discrete Cre expression patterns demonstrate that topologically distinct populations of CLA neurons provide dense inputs onto the RSC and M1. In contrast, BLA-projecting neurons with the strongest Cre expression were found in the upper layers of the insula, while only a small number of faintly labeled neurons were observed in the region immediately surrounding the vCLA core (Figure 1C, Cre). This region between the vCLA core and L5b of the AI is also referred to as the vCLA shell (Smith et al., 2018; Marriott et al., 2021).

To observe potential co-projection networks from CLA neurons going to different initial targets, we relied on the expression of Cre to label projection-defined CLA neurons with mCherry. The CLA-specific Cre expression from the RSC and M1 initial targets allowed us to easily limit mCherry expression to the CLA neurons (Figures 1A,B, mCherry). However, when the BLA was the initial target, the large number of Cre-labeled neurons in the insula made CLA-specific labeling a challenge (Figure 1C, Cre). We successfully limited mCherry labeling to BLA-projecting CLA neurons by specifically targeting injection of the Cre-dependent vector away from the insula, thereby avoiding Cre-dependent vector transfection in Cre-expressing neurons in the deep layers of the insula (see “Materials and Methods” Section). This yielded strong mCherry expression in vCLA shell neurons (Figure 1C). This is presumably a consequence of the enzymic nature of Cre, which catalyzes strong mCherry expression from a small amount of Cre. For the BLA initial target, Cre-dependent mCherry labeling revealed a large number of neurons in the vCLA shell that project to the BLA (Figure 1C, mCherry). This indicates that many CLA neurons also project to the BLA. Moreover, these BLA-projecting neurons clearly reside in a distinct zone of the CLA compared to neurons projecting to the M1 or the RSC.

Using the above targeting strategies, we successfully limited mCherry expression to CLA neurons, and demonstrated that the somata of CLA neurons projecting to the RSC (retRSC), BLA (retBLA), or M1 (retM1) are clearly located in distinct topological zones within the CLA complex (Figure 1). These results support the notion of functional modularization in the CLA, as CLA neurons projecting to different regions seem to segregate into distinct anatomical modules.

**Figure 2:**
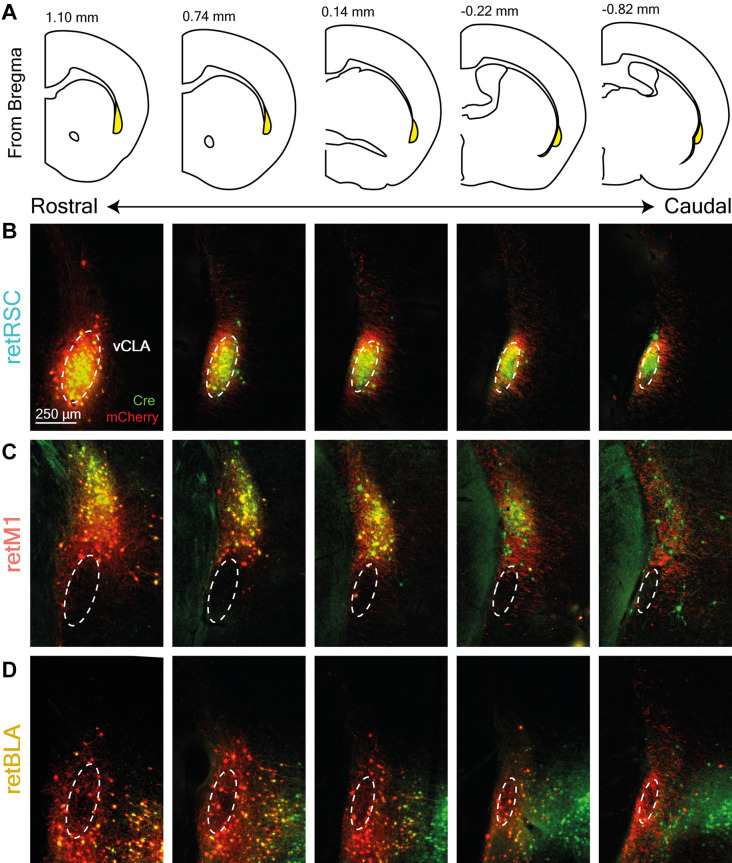
Topological zones in the CLA are maintained throughout the rostral-caudal axis. **(A)** Diagram of coronal sections corresponding to CLA images shown in **(B–D)**. The CLA is highlighted in yellow and the distance from the bregma is indicated at the top. **(B–D)** Examples of retrograde Cre (green) and conditional mCherry (red) expression across the entire rostral-caudal extent of the CLA (columns). Each row corresponds to a different initial target: **(B)** RSC-projecting (retRSC), **(C)** BLA-projecting (retBLA), and **(D)** M1-projecting (retM1). Dotted white ellipses demarcate the ventral CLA core (vCLA) based on parvalbumin staining (not shown).

We also examined the distribution of Cre and mCherry expression in the CLA across the rostral-caudal axis to determine whether these topological zones are restricted to particular rostral-caudal segments (Figures 2B,C). PV staining was again used to delineate the vCLA core (Figure 2, dotted white ellipses). The relative distribution of fluorophore expression was maintained throughout the entire rostral-caudal extent of the CLA, suggesting that the CLA is primarily organized in the coronal plane.

The distinct projection-based segregation of CLA topology is potentially in conflict with earlier reports of CLA neurons that project to multiple regions (Kitanishi and Matsuo, 2017; Zingg et al., 2018; Peng et al., 2021). To reconcile these two views, we next examined the projection networks of these three CLA populations by scanning the brain for axons labeled with mCherry to identify all their axonal targets.

### RSC-Projecting CLA Neurons Project Widely Throughout the Brain

**Figure 3:**
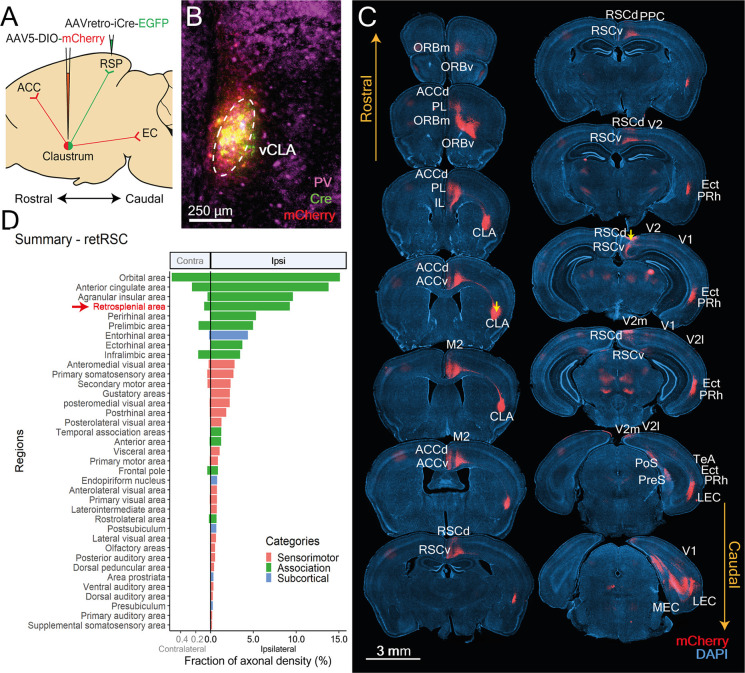
CLA neurons projecting to RSC (retRSC) reside within the ventral CLA core and co-project mainly to higher cortical targets. **(A)** Double-virus injection strategy used to label CLA neurons projecting to the RSC and its axon arborizations. **(B)** CLA-specific expression of Cre and mCherry. vCLA core boundaries (white dotted ellipse) are demarcated based on parvalbumin (PV) antibody staining. **(C)** Mapping of retRSC neuron axons; images show mCherry expression (red) within DAPI-stained (blue) coronal sections. Yellow arrows denote viral injection sites. CLA neurons projecting to RSC also project to several other higher-order cortical structures. Labels indicate regions receiving CLA axons. PL, Prelimbic cortex; ORBm, ORBv, Orbital frontal cortex medial, ventral; ACCd, ACCv, Anterior cingulate cortex dorsal, ventral; IL, Infralimbic cortex; M1, M2, Primary and secondary motor cortex; RSCd, vRSCv, Retrosplenial cortex dorsal, ventral; PPC, Posterior Parietal Cortex; V1, V2, V2 m, V2l, primary and secondary visual cortex, medial, lateral; Ect, Ectorhinal cortex; Prh, Perirhinal cortex; TeA, Temporal association cortex; PreS, Presubiculum; PoS, Postsubiculum; ENTl, ENTm, Entorhinal cortex lateral, medial. **(D)** Mean fraction of axonal density for all innervated regions, sorted by density. Red arrow and text indicate retrograde Cre origin (RSC). Orbital, anterior cingulate and agranular insula had heavier innervation compared to RSC. Colors represent region categories: Association—green, Sensorimotor—pink, and Subcortical—Blue. Note different ranges for contralateral and ipsilateral values.

The high specificity of CLA labeling following injection of retrograde AAV vectors in the RSC has frequently been used to target the CLA *via* the expression of Cre-recombinase (Jackson et al., 2018; Zingg et al., 2018). Using such a viral-based Cre-lox strategy (Figure 3A), we mapped out axonal networks of the RSC-projecting CLA neurons. Consistent with prior studies (Jackson et al., 2018; Zingg et al., 2018), injection of retrograde AAVs into the RSC yielded a highly specific expression of EGFP—expressed along with Cre—within the PV-rich vCLA core but not the surrounding insula cortex (Figure 3B). Cre-dependent mCherry expression was also restricted to retRSC neurons that were clearly contained within the vCLA core (Figure 3B, mCherry).

To determine whether retRSC neurons also project to other cortical targets, we examined the distribution of their mCherry-labeled axons (Figure 3C). Strongly labeled axons were found in numerous cortical regions spanning the entire anterior-posterior extent of the brain, indicating that retRSC neurons project to many other regions beyond the RSC (Figure 3C). We presume that these projections reflect synaptic input coming from the CLA. Axon labeling was also clearly stronger in the ipsilateral hemisphere (Figure 3D), indicating that retRSC network mainly influences the activity of ipsilateral structures.

**Figure 4:**
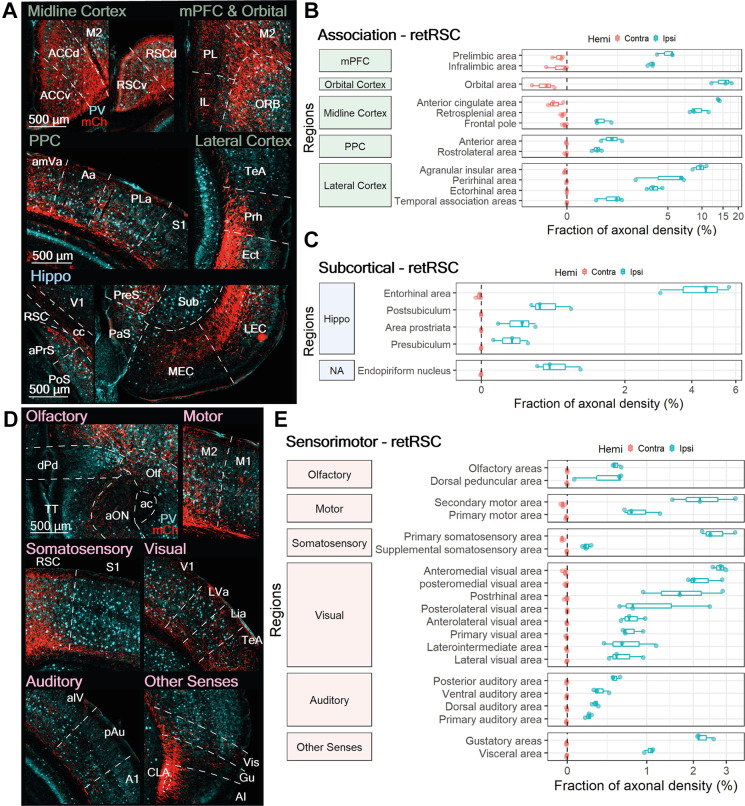
RSC projecting neurons heavily innervate other association-related regions, some subcortical targets, and numerous sensorimotor regions. **(A,D)** Representative images from ipsilateral regions innervated by RSC-projecting CLA neurons (retRSC): **(A)** association, subcortical, and **(D)** sensorimotor. mCherry labeled axon fibers in red (mCh) and PV expression in cyan. **(B,C,E)** Quantification of regions innervated by retRSC in the contralateral (red) and ipsilateral (blue) hemispheres. Innervation from retRSC is largely ipsilateral, except for the orbital area. Axon fiber density from each region is represented as a fraction of whole brain axon density and is on a logarithmic scale. **(B)** retRSC preferentially innervates the orbital area, anterior cingulate cortex, and agranular insula over other cortical regions. **(C)** retRSC neurons also innervate several subcortical regions, with most related to the hippocampal formation. **(E)** retRSC neurons preferentially innervate secondary motor, primary somatosensory, secondary visual, and gustatory regions. Region abbreviations for **(A)**: ACCv, ACCd, Anterior cingulate cortex ventral, dorsal; M2, Secondary motor cortex; RSCv, RSCd, Retrosplenial cortex ventral, dorsal; IL, Infralimbic cortex; PL, Prelimbic cortex; ORB, Orbital area; amVa, Antereomedial visual area; Aa, Anterior area; PLa, Posterolateral visual area; S1, Primary somatosensory cortex; TeA, Temporal association cortex; Prh, Perirhinal cortex; Ect, Ectorhinal cortex; aPrs, Area prostriata; PoS, Postsubiculum; cc, Corpus callosum; V1, primary visual cortex; PaS, Parasubiculum; PreS, Presubiculum; MEC, LEC, Medial, Lateral entorhinal cortex; Sub, Subiculum. Region abbreviations for **(D)**: dPd, dorsal peduncular area; TT, Taenia tecta; aON, anterior olfactory nucleus; ac, anterior commissure; Olf, Olfactory areas; M2, Secondary motor cortex; M1, Primary motor cortex; RSC, Retrosplenial cortex; S1, Primary somatosensory cortex; V1, Primary visual cortex; LVa, Lateral visual area; Lia, Laterointermediate area; TeA, Temporal association area; alV, anterolateral visual area; pAu, posterior auditory area; A1, Primary auditory area; CLA, Claustrum; Vis, Visceral area; Gu, Gustatory area; AI, Agranular Insula.

To quantify the distribution of axons expressing mCherry, fluorescence images were measured by adapting processes from the QUINT workflow (Yates et al., 2019). Despite using the RSC as the initial target, several brain regions received stronger innervation compared to the RSC. To better understand differences in innervation between downstream targets, brain regions targeted by RSC-projecting neurons were grouped into three broad categories—association, sensorimotor or subcortical—and further divided into functional groups within each category (Figure 4).

#### RSC-Projecting Neurons Heavily Innervate Many Association Cortices

Most ipsilateral association regions accounted for between 5% and 20% of axonal density across the brain (Figure 4B). In addition to the RSC, retRSC neurons also strongly innervated several ipsilateral midline, frontal, and lateral cortices: the ORB, prelimbic cortex (PL), infralimbic cortex (IL), ACC, AI, perirhinal (Prh), and ectorhinal cortex (Ect; Figures 4A,B). Moderate axonal densities were also observed in the ipsilateral FrP, temporal association cortex (TeA), and posterior parietal cortices (PPC—Aa, rlA; Figures 4A,B). Although some contralateral association regions—medial prefrontal cortex (mPFC), orbital area (ORB), anterior cingulate cortex (ACC), frontal pole (FrP), and agranular insula (AI)—also had moderate to weak innervation, their axonal densities were orders of magnitude lower than their ipsilateral counterparts. Thus, although CLA neurons projecting to the RSC also project to several other association-related cortical regions, the extent of innervation varies depending on the target.

#### RSC-Projecting Neurons Moderately Innervate Some Subcortical Regions

In addition to ipsilateral association regions, several ipsilateral subcortical regions also received moderate innervation from retRSC neurons; this ranged from 0.4% to 4.5% of total axon density across the brain. Most of the innervated subcortical regions were associated with the hippocampal formation: entorhinal (EC), postsubiculum (PoS), area prostriata (aPrS), and presubiculum (Pre; Figures 4A,C). retRSC innervations appeared to be denser in the lateral entorhinal cortex (LEC) than the medial entorhinal cortex (MEC; Figure 4A, bottom right panel). This suggests that the retRSC network may be more involved with the processing of objects, attention, and motivation—which are functions associated with the LEC—rather than space, which is associated with the MEC (Witter et al., 2017). In addition to the hippocampal formation, retRSC neurons also moderately innervated the endopiriform nucleus (En), located directly beneath the CLA (not shown in Figure 4). Despite the proximity of the En to the CLA, CLA innervation of the En was only 0.78 ± 0.3% of total axonal density, suggesting that these two subcortical structures may only be weakly connected.

#### RSC-Projecting Neurons Innervate All Sensorimotor Modules

At least one region belonging to each sensorimotor module (i.e., Olfactory, Motor, Somatosensory, Visual, Auditory, Other Senses) received innervation from retRSC neurons. This sensorimotor innervation again was mostly ipsilateral, with only sparse innervation observed in some contralateral olfactory and secondary motor areas (Figure 4E, Olfactory and Motor, red). Moderate ipsilateral innervation was observed in the secondary motor (M2), primary somatosensory (S1), gustatory (Gu), and visceral (Vis), as well as some secondary visual cortices: anteromedial visual, posteromedial visual, and postrhinal area (amV, pmV, PoRh; Figures 4D,E).

Higher-order sensorimotor regions receiving moderate input (M2 and some secondary visual cortices) were innervated throughout all their layers, while other sensorimotor regions with moderate input (Gu, Vis, S1) were mainly innervated in the deeper layers (Figure 4D, Motor, Somatosensory, and Other Senses). These differences in layer-specific innervation suggest that the retRSC network may be providing general inputs to higher-order sensorimotor regions, compared to the more precise input provided to the deep layers of primary sensorimotor regions. Other sensorimotor modules, such as olfactory and auditory structures, were also weakly innervated (<1% of total axonal density) in some sub-regions (Figures 4D,E). This suggests that the retRSC network provides only a minor influence on other sensorimotor modules.

In summary, retRSC neurons reside in the vCLA core and project not only to the retrosplenial cortex but also to numerous association, sensorimotor and subcortical regions. Consistent with previous reports (Wang et al., 2017; Zingg et al., 2018), the highest degree of innervation was concentrated in the association regions (Figure 3D). We also identified several novel sensorimotor regions that received moderate to weak innervation from retRSC neurons; most notably in S1, M2, Gu, and some secondary visual cortices. Although the retRSC network is positioned to influence all sensorimotor modules, these results indicate an apparent preference for visual, motor, gustatory and somatosensory modules over olfactory and auditory modules. Lastly, weak innervation was also identified in some subcortical regions associated with the hippocampal formation and the En, indicating that the retRSC network may provide some input to these subcortical structures. These results align well with previously reported findings, validating our experimental approach, and have identified new features of CLA output networks.

### M1-Projecting CLA Neurons Reside in the Dorsal CLA and Project to Few Other Targets

Studies of latexin-defined CLA neurons (Watakabe et al., 2014; Orman, 2015; Dillingham et al., 2017) and additional studies based on cell-specific targeting methods (Atlan et al., 2018; Fodoulian et al., 2020; Narikiyo et al., 2020) have indicated neurons that apparently reside in both the dCLA and the vCLA. Comparing the projection targets of retM1 and retRSC—two topologically-distinct CLA neuron populations residing in the dCLA and vCLA core, respectively—provides us with an opportunity to better understand the results obtained from more inclusive CLA definitions.

**Figure 5:**
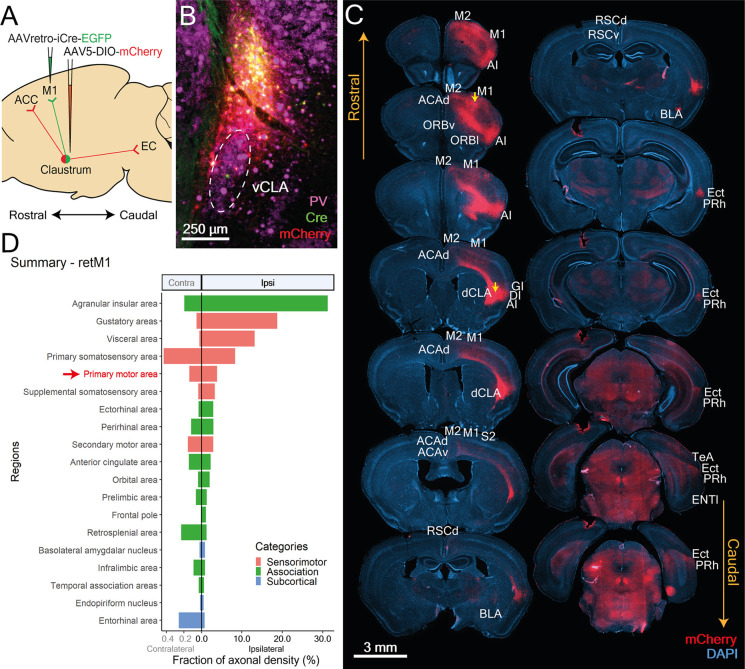
CLA neurons projecting to M1 localize in the dorsal CLA and co-project to sensorimotor-related cortical regions. **(A)** Illustration of double virus injection strategy used to label CLA neurons projecting to the M1 and their axons. **(B)** CLA-specific expression of Cre and mCherry based on experiment shown in **(A)**. vCLA core boundaries (white dotted ellipse) are demarcated based on parvalbumin (PV) antibody staining. **(C)** Mapping of axon collateral from M1-projecting CLA neurons. Raw images of mCherry expression against DAPI stained coronal sections. Yellow arrows denote viral injection sites. Regions receiving axons are labeled. M1, M2, Primary and secondary motor cortex; AI, Agranular insula; ACCd, Anterior cingulate cortex dorsal; ORBv, ORBl, Orbital cortex ventral, lateral; GI, DI, Granular, Dysgranular Insula; dCLA, dorsal claustrum; RSCd, vRSCv, Retrosplenial cortex dorsal, ventral; S2, Secondary somatosensory cortex; BLA, Basolateral amygdala; Ect, Ectorhinal cortex; Prh, Perirhinal cortex; TeA, Temporal association cortex; LEC, Lateral entorhinal cortex. **(D)** Mean fraction of axonal density for all innervated regions, sorted by labeling strength. Red arrow and text identifies retrograde Cre origin: M1. Agranular insula, gustatory, visceral, and primary somatosensory cortices had heavier innervation compared to M1. Colors represent region categories: Association—green; Sensorimotor—pink; and Subcortical—Blue. Note different ranges for contralateral and ipsilateral values.

Employing the same viral strategy used for retRSC, we retrogradely tagged CLA neurons projecting to M1 with Cre, and selectively expressed mCherry to investigate its output networks (Figure 5A). retM1 neurons were found to mostly cluster in the dCLA with a small number of neurons in the vCLA shell (Figure 5B). To determine whether retM1 neurons also send axons to cortical targets beyond M1, we again examined coronal slices across the entire rostral-caudal extent of the rodent brain (Figure 5C, mCherry). Labeled axons were largely restricted to ipsilateral motor-related cortices, with varying axonal densities in a few other ipsilateral cortical targets. Subsequent quantification revealed that retM1 neurons also project to several other sensorimotor, association, and subcortical regions in both hemispheres (Figure 5D), although outputs were again heavier on the ipsilateral side. The degree of axonal branching for retM1 neurons was also more restricted compared to that of retRSC, indicating that retM1 may have more focused outputs. Targeted brain regions were next grouped into sensorimotor, association, or subcortical for deeper analysis.

#### M1-Projecting CLA Neurons Project to Other Sensorimotor Regions

**Figure 6:**
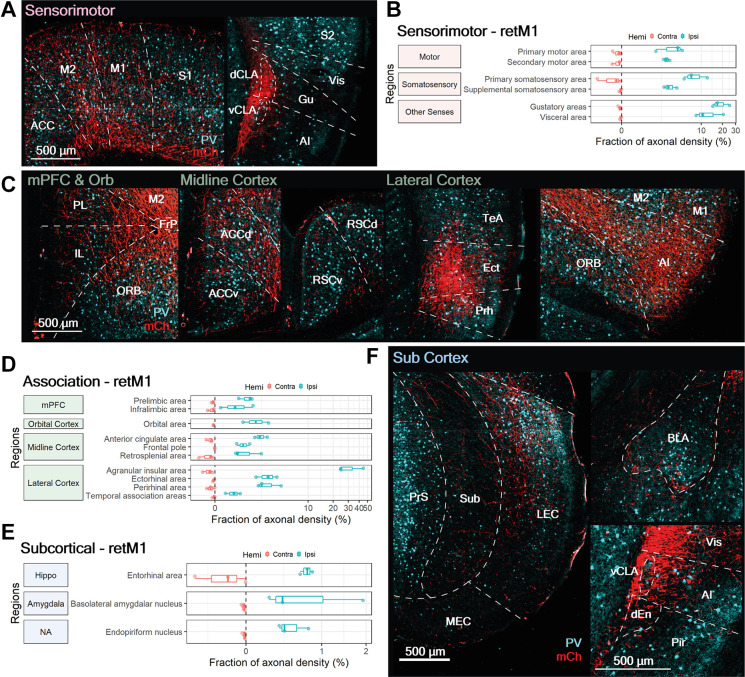
M1-projecting CLA neurons heavily innervate select sensorimotor cortices, moderately innervate association-related regions and sparsely innervate subcortical regions. **(A,C,F)** Representative images from ipsilateral regions innervated by M1-projecting CLA neurons: **(A)** sensorimotor, **(C)** association, and **(F)** subcortical. mCherry labeled axons in red (mCh) and PV expression in cyan. **(B,D,E)** Quantification of regions innervated by M1-projecting CLA neurons in the contralateral (red) and ipsilateral (blue) hemispheres for **(B)** sensorimotor, **(D)** association, and **(E)** subcortical. Axonal density from each region is represented as a fraction of whole brain density; note that logarithmic scale. Region abbreviations: ACC, Anterior cingulate cortex; M2, M1, Secondary, primary motor cortex; S1, S2, Primary, secondary somatosensory cortex; dCLA, vCLA, dorsal, ventral claustrum; Vis, Visceral area; Gu, Gustatory area; AI, Agranular Insula; PL, Prelimbic cortex; IL, Infralimbic cortex; ORB, Orbital area; FrP, Frontal pole; ACC, ACCd, ACCv, Anterior cingulate cortex, dorsal, ventral; FrP, Frontal pole; RSCd, RSCv, Retrosplenial cortex dorsal, ventral; TeA, Temporal association area; Ect, Ectorhinal cortex; Prh, Perirhinal cortex; PrS, Presubiculum; Sub, Subiculum; LEC, MEC, Lateral, medial entorhinal cortex; BLA, Basolateral amygdala; vCLA, ventral claustrum; dEn, dorsal endopiriform nucleus; Vis, Visceral cortex; Pir, Piriform cortex.

As a whole, sensorimotor regions accounted for the largest proportion of retM1 axon density across the three categories. Sensorimotor innervation by retM1 neurons was largely ipsilateral, with only weak innervation in the contralateral M1, M2 and S1 (Figure 6B, Contra). Besides the ipsilateral M1, retM1 neurons also projected heavily to other ipsilateral sensorimotor regions such as the M2, S1, S2, Gu, and Vis (Figures 6A,B; Ipsi).

Compared to M1, where the retrograde Cre virus was injected, axonal density was much higher in Gu, Vis, and S1. Although a fraction of measured axonal fluorescence in the Gu and Vis could result from the proximity of the dCLA, where retM1 cell bodies reside, this potential confound would be restricted to the deep layers of the Gu and Vis. Nonetheless, substantial innervation was also observed in the superficial layers of both Gu and Vis (Figure 6A, right panel), verifying strong connections from the dCLA to both regions. Among the sensorimotor cortices related to somatic and visceral function, retM1 axonal fibers appear to extensively innervate all cortical layers (Figure 6A, right panel); this suggests region-wide influence, as opposed to more precise, layer-specific control.

#### M1-Projecting CLA Neurons Project to Specific Association-Related Cortical Regions

In addition to innervating sensorimotor structures, retM1 neurons also project to several association-related cortical regions. Association cortex innervation by retM1 neurons was again largely ipsilateral with only weak innervation of some contralateral counterparts, such as the mPFC, ACC, RSC, AI, and Prh (Figure 6D, Contra). Exceptionally dense innervation was found in the ipsilateral rostral agranular insula cortex (>30%; AI, Figure 6C, right-most panel and Figure 6D), suggesting that the major output of retM1 neurons may be the ipsilateral rostral AI. Other ipsilateral cortical regions, such as the ORB, midline cortices (ACC, FrP, RSC), Ect, and Prh, also received moderate degrees of innervation from retM1 neurons (Figures 6C,D). Sparse innervation was also observed in the ipsilateral medial prefrontal cortex (PL, IL), and TeA. The presence of axonal inputs to these association-related cortical regions suggests that, despite a strong focus on sensorimotor outputs, retM1 neurons residing in the dorsal CLA may also influence higher-order cortices.

#### Subcortical Targets of M1-Projecting CLA Neurons

Within the subcortical category, only three regions were weakly innervated by retM1 neurons. Subcortical innervation was mostly on the ipsilateral side, with some innervation found in the contralateral EC that was still less dense than on the ipsilateral side (Figure 6E, Contra). retM1 neuron innervation of the EC, basolateral amygdala (BLA), and En accounted for less than 1% of axonal density across the brain, indicating that retM1 neurons only lightly innervate these regions. Labeled axons in the entorhinal area were largely restricted to the LEC with decreasing fiber densities toward the MEC (Figure 6F, left panel). This indicates that the retM1 network—similar to the network of retRSC neurons—may also be more involved with the processing of objects, attention, and motivation rather than space (Witter et al., 2017). Although retM1 connections to regions within the subcortical category were sparse, retM1 axons covered the entire extent of each region (Figure 6F). This may indicate that retM1 neuron output broadly influences these subcortical regions.

In summary, we established that dCLA neurons project to multiple targets. retM1 neurons send axons to several sensorimotor, cortical, and subcortical regions across the brain, albeit with more target focus in comparison to neurons in the vCLA core (Figure 2D). Similar to the vCLA core neurons, dCLA neurons mainly project to the ipsilateral hemisphere. However, in contrast to vCLA core neurons, dCLA neurons project heavily to both sensorimotor and cortical regions, with a slight preference for sensorimotor cortices.

### BLA-Projecting Neurons Project to Other Subcortical Targets

We also examined the output networks of BLA-projecting CLA neurons, which reside in the vCLA shell. Amongst the three projection-defined CLA neuron populations we have examined, CLA neurons projecting to the basolateral amygdala (retBLA) are the least investigated and most controversial. The CLA-to-BLA connection has been proposed to be an important component of CLA function, potentially mediating the exchange of salience-related information between the two structures (Smith et al., 2019, 2020). However, it is unknown whether these neurons also project to multiple regions and whether they exhibit unique projection patterns. Further, although there are genetic differences between vCLA shell neurons and those in the insula (Watson and Puelles, 2017; Smith et al., 2018; Binks et al., 2019), it has been proposed that neurons residing in this shell-like region belong to deep layers of the insula (Mathur, 2014). Because retBLA neurons residing in this shell-like region extend around the entire perimeter of the vCLA core (Figure 2D), rather than only in the area between the vCLA core and insula, we consider them to be vCLA shell neurons.

**Figure 7:**
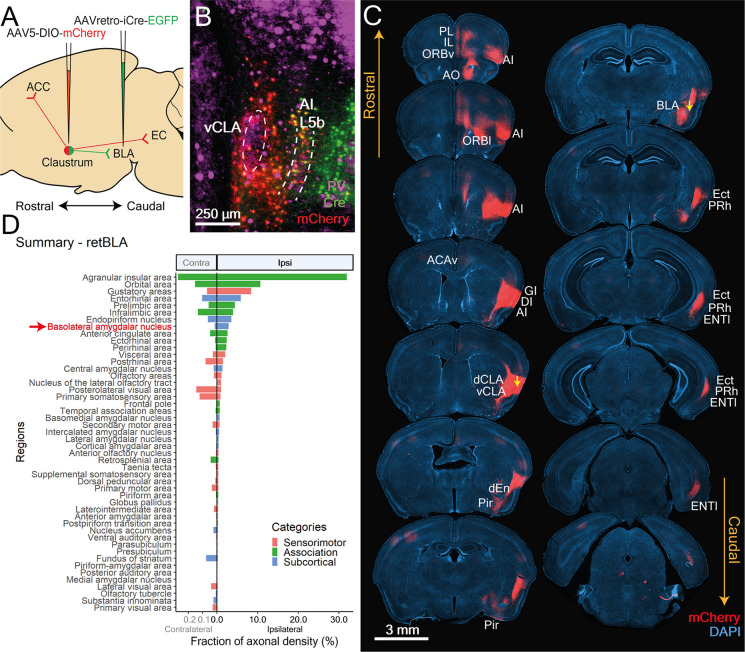
CLA neurons projecting to the BLA reside in a shell-like region surrounding the vCLA core and co-project to limbic-related cortices. **(A)** Illustration of double virus injection strategy used to label CLA neurons projecting to the BLA and its axon arborizations. **(B)** CLA-specific expression of Cre and mCherry based on experiment shown in **(A)**. vCLA core boundaries (white dotted ellipse) and layer 5b of the anterior insula (white dotted lines) are demarcated based on parvalbumin (PV) antibody staining. **(C)** Mapping of axon collateral from BLA-projecting CLA neurons. Raw images of mCherry expression against DAPI stained coronal sections. Yellow arrows denote viral injection sites. CLA neurons projecting to BLA also project to several other limbic-related cortices. Regions receiving arborizations are labeled. PL, Prelimbic; IL, Infralimbic; AO, anterior olfactory nucleus; AI, Agranular insula; ORBv, ORBl, Orbital cortex ventral, lateral; ACCv, Anterior cingulate cortex ventral; GI, DI, Granular, Dysgranular Insula; dCLA, vCLA, dorsal, ventral claustrum; dEn, dorsal endopiriform nucleus; Pir, Piriform cortex; BLA, Basolateral amygdala; Ect, Ectorhinal cortex; Prh, Perirhinal cortex; LEC, Lateral entorhinal cortex; Sub, Subiculum. **(D)** Mean fraction of axonal density for all innervated regions sorted by strength. Red arrow and text identify retrograde Cre origin: BLA. Several regions had heavier innervation compared to the BLA. These include the agranular insula, orbital, gustatory, entorhinal, prelimbic, and infralimbic cortices as well as the endopiriform nucleus. Colors represent region categories: Association—green, Sensorimotor—pink, and Subcortical—Blue. Note different ranges for contralateral and ipsilateral values.

Using the same viral-based targeting strategy employed for retRSC and retM1, we labelled BLA-projecting neurons residing in the vCLA shell with mCherry (Figures 7A,B). Due to the proximity of the AI to the CLA, we inevitably labeled some AI neurons that also project to the BLA. While this could have confounded our efforts to track retBLA axons emerging from the CLA, it was not a problem because mCherry labeling of AI neurons was clearly much weaker than that of CLA neurons (Figure 7B). In addition, based on the proportion of labeled regions, a majority of the downstream regions seem to be unique to CLA neurons, rather than AI neurons: we did not find strong labeling in the thalamus and striatum (Figure 7D), which are known to receive extensive AI input (Gehrlach et al., 2020). Hence the axonal networks that we observed are likely to uniquely represent, or at least are very heavily biased toward, the vCLA shell neuron network.

To determine whether BLA-projecting CLA neurons extend to other regions, mCherry expression was examined throughout the entire brain (Figure 7C). In addition to the expected projections to the BLA, dense mCherry labeling was also identified in many ipsilateral limbic-related cortical regions. Subsequent quantification revealed a substantial number of regions receiving innervation from retBLA neurons that were heavier than that of the BLA (Figure 7D). This suggests that the BLA is not the major output target of vCLA shell neurons. In addition, moderate to sparse innervation was also identified in numerous regions spanning all three broad categories: association cortex, sensorimotor cortex, and subcortical (Figure 7D). This widespread innervation of regions throughout the brain indicates that retBLA neurons may influence a large number of brain regions in concert.

#### BLA-Projecting CLA Neurons Also Project to Association-Related Cortical Regions

**Figure 8:**
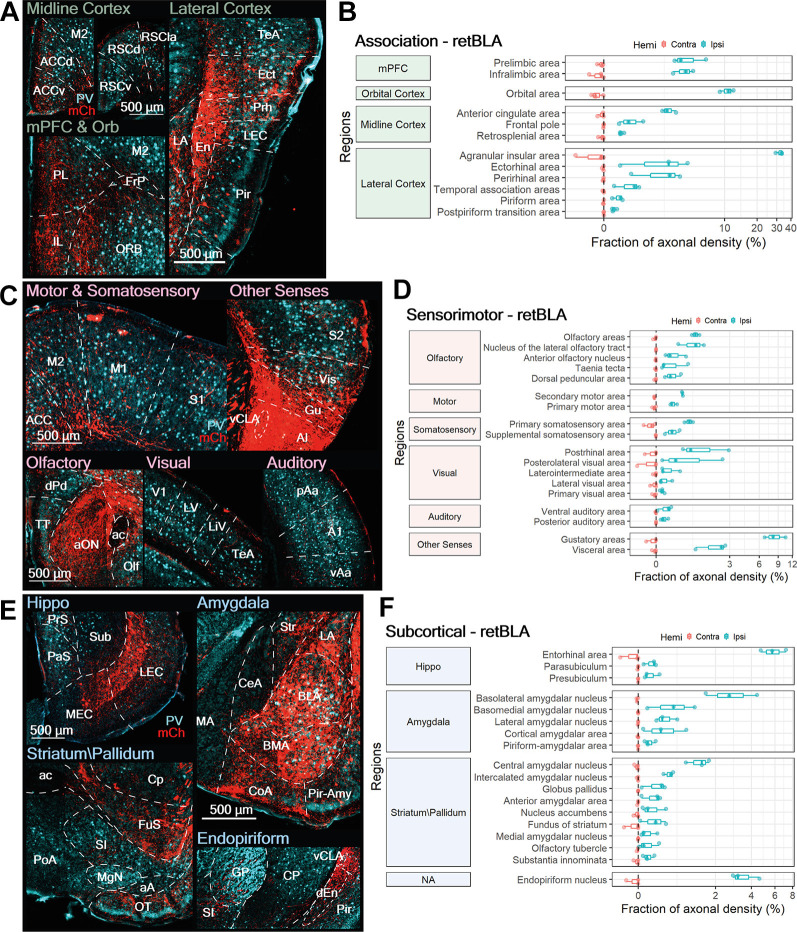
BLA-projecting CLA neurons project to numerous limbic-related sensorimotor, association, and subcortical targets. **(A,C,E)** Representative images from ipsilateral regions innervated by BLA-projecting CLA neurons: **(A)** association, **(C)** sensorimotor, and **(E)** subcortical. mCherry labeled axon fibers in red (mCh) and PV expression in cyan. **(B,D,F)** Quantification of regions innervated by BLA-projecting CLA neurons in the contralateral (red) and ipsilateral (blue) hemispheres for **(B)** association, **(D)** sensorimotor, and **(F)** subcortical. Axon density from each region is represented as a fraction of whole brain axon density; note logarithmic scale. Region abbreviations: ACC, ACCd, Anterior cingulate cortex, dorsal; M2, M1, Secondary, primary motor cortex; RSCv, RSCd, RSCla, Retrosplenial cortex ventral, dorsal, lateral; PL, Prelimbic cortex; IL, Infralimbic cortex; FrP, Frontal Pole; ORB, Orbital area; dEn, En, dorsal, Endopiriform nucleus; TeA, Temporal association area; Ect, Ectorhinal cortex; Prh, Perirhinal cortex; Pir, Piriform cortex; S1, S2, Primary, secondary somatosensory cortex; Vis, Visceral area; Gu, Gustatory area; AI, Agranular Insula; vCLA, ventral claustrum; TT, Taenia tecta; dPd, Dorsal peduncular area; aON, Anterior olfactory nucleus; ac, anterior commissure; Olf, Olfactory area; V1, Primary visual cortex; LV, Lateral visual area; LiV, Laterointermediate area; pAa, vAa, A1, Posterior, ventral, primary auditory area; PrS, Presubiculum; PaS, Parasubiculum; Sub, Subiculum; LEC, MEC, Lateral, medial entorhinal cortex; Str, Striatum; MA, CeA, LA, BLA, BMA, Medial, central, lateral, basolateral, basomedial amygdala nucleus; CoA, Pir-Amy, Cortical, Piriform- amygdala area; Cp, Caudate putamen; FuS, Fundus of striatum; SI, Substantia inominata; PoA, Posterior amygdala nucleus; MgN, Magnocellular nucleus; aA, anterior amygdala area; OT, Olfactory tubercle; GP, Globus pallidus.

Several frontal and lateral association cortices were innervated by CLA neurons projecting to the BLA (retBLA). Innervation of association regions from retBLA neurons was mainly ipsilateral, with only light contralateral projections to the mPFC, ORB, ACC, RSC, and AI (Figures 8A,B). Similar to retM1 projections, retBLA innervation of the ipsilateral AI was exceptionally strong (AI; 31.9 ± 2.2%). Measurement of axonal projections to the AI may be overestimated due to the fact that the digital atlas defines the vCLA shell as being part of the AI. Therefore, the somatic expression of mCherry in vCLA shell neurons overlaps with fibers in these AI layers. Nevertheless, high axonal densities were also identified in AI regions far from the injection site (Figure 8C, rostral AI), indicating that retBLA neurons do heavily innervate the AI.

Beyond innervation of the AI, retBLA neurons also heavily innervated several ipsilateral association regions: the ORB, mPFC (PL, IL), ACC, Ect, and Prh (Figures 8A,B). Moderate to sparse innervation was also observed in the RSC, FrP, TeA, Pir, and postpiriform transition area (Figures 8A,B). Innervation of these association cortices indicates that CLA neurons that project to the BLA also project to multiple association-related cortices, similar to CLA neurons in the dCLA and the vCLA core.

#### BLA-Projecting CLA Neurons Project to Select Sensorimotor Regions

In addition to innervating association areas, retBLA neuron axons were also identified in several sensorimotor regions. Such sensorimotor innervation was largely ipsilateral, with only weak innervation of the contralateral S1, secondary visual cortex (V2), Gu, and Vis (Figure 8D). Of the ipsilateral sensorimotor regions, the Gu accounted for the largest fraction of axonal projections. This indicates that the major sensorimotor output from retBLA neurons is the Gu (Figures 8C, Figures 7D, Other Senses). A few other ipsilateral sensorimotor regions, namely the Vis, Olf, nucleus of lateral olfactory tract, PoRh, and posterolateral visual area also received moderate innervation (Figures 8C,D). Thus, retBLA neurons appear to preferentially target visceral areas, as well as select olfactory, and secondary visual areas. Weak innervation was also observed for both primary and secondary sensorimotor regions of all sensorimotor modalities, aside from the primary auditory cortex (Figures 8C,D). This indicates that retBLA neurons could participate in all sensorimotor modalities. For all sensorimotor regions, retBLA innervation seemed to span the entire cortical column, with a sparse distribution of axons throughout all cortical layers (Figure 8C). The presence of axons in all layers suggests that CLA neurons projecting to the BLA are positioned to broadly influence all innervated sensorimotor regions.

#### BLA-Projecting CLA Neurons Innervate Many Limbic-Related Subcortical Regions

Similar to the innervation of sensorimotor areas, axonal projections to areas within the subcortical category accounted for approximately 20% of the total distribution of retBLA neuron axons. The majority of subcortical innervation was ipsilateral, with only weak innervation of the contralateral EC, fundus of striatum, and En. Besides the ipsilateral BLA—where retrograde Cre was injected—particularly heavy ipsilateral innervation was observed in the EC and En (Figures 8E,F). The heavy innervation of the EC and En indicates that retBLA neurons could preferentially influence these limbic-related structures compared to other subcortical targets. Innervation of the EC was concentrated in the deep layers of both LEC and MEC (Figure 8E, Hippo), indicating that the retBLA network may influence both the processing of objects, attention, and motivation, as well as the processing of space (Witter et al., 2017). Moderate to low innervation was also found for numerous other subcortical structures associated with the hippocampal formation, amygdala, striatum, and pallidium (Figures 8E,F). These results indicate that CLA neurons projecting to the BLA also innervate numerous other limbic-related subcortical structures. Thus, retBLA neurons are positioned to influence several limbic structures in concert, albeit to varying extents.

Our results from axonal mapping of retBLA neurons establish for the first time that vCLA shell neurons project to multiple targets across the brain. Similar to the other CLA projection neurons (retM1 and retRSC), retBLA neurons also project heavily to association regions. However, retBLA outputs were more concentrated in limbic-related regions, suggesting that modality preferences do differ between the three projection-defined CLA populations.

### Topology-Defined CLA Networks Differ but Overlap to Varying Extents

**Figure 9:**
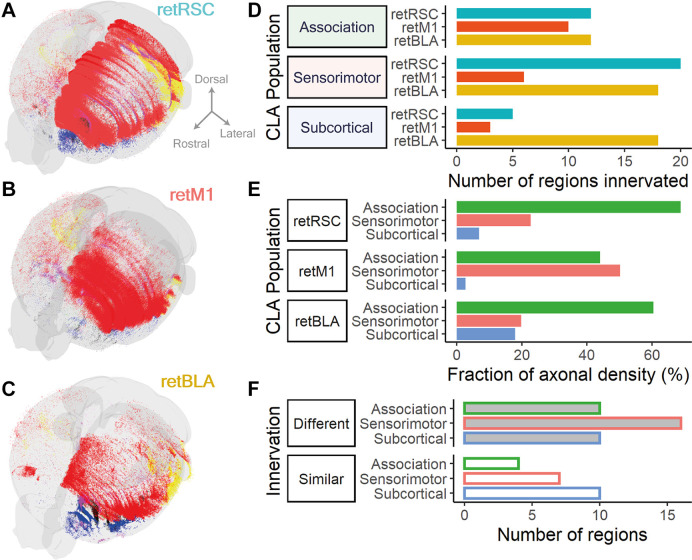
CLA neurons projecting to RSC, BLA, and M1 form different projection distributions and differ most heavily in sensorimotor innervation. **(A–C)** 3D reconstruction of fiber densities as point clouds; colors represent different broad region categories: Cortex, red; Olfactory, blue; and Hippocampal formation, yellow. **(A)** From RSC-projecting CLA neurons (retRSC). **(B)** From M1-projecting CLA neurons (retM1). **(C)** From BLA-projecting CLA neurons (retBLA). **(D)** Fraction of axonal density from each CLA projection population split into association (green), sensorimotor (red), and subcortical (blue) categories.The distribution of axonal densities across the different categories differed between the three CLA populations. **(E)** Number of regions innervated by each CLA projection population grouped by broad region categories: Association, sensorimotor and subcortical. Only regions with mean axonal density >0.002 of the total were considered to be innervated. Colors represent different CLA populations: retRSC in teal, retM1 in orange, retBLA in yellow. The number of innervated regions was similar for association cortices between all three CLA populations but differed for the sensorimotor and subcortical categories. **(F)** The number of regions in each broad region category that had different (top, shaded bar) or similar (bottom, open bar) innervation by all three CLA populations. The difference in innervation was determined by statistical testing using ANOVA to compare axonal densities from each CLA neuron population to each innervated brain region. Colored border represents different broad region categories: Association in green, sensorimotor in red, and subcortical in blue. The number of regions that had different innervation between CLA populations was higher than the number of regions that had similar innervation for association and sensorimotor cortices, but were equal in number for the subcortical category.

Having quantified the axonal distributions of all three projection-defined CLA networks, we next compared these topology-separated CLA networks to each other. As output networks from all three CLA neuron populations were largely ipsilateral, our analyses focused on ipsilateral connections. 3D reconstruction of axonal networks from each CLA neuron type exemplified differences in their projection preferences (Figures 9A–C). Fiber densities are represented as clouds of points and are colored based on their broad atlas categories: red for cortex, blue for olfactory, and yellow for the hippocampal formation. Major differences in projection network topology—such as the preference of retRSC for midline cortical regions—were readily apparent (Figures 9A–C). However, sparser innervation also covered many overlapping cortical areas, suggesting these CLA networks were not completely separate.

Both the network span (number of regions; Figure 9D) and network profile (fraction of axonal density; Figure 9E) associated with the different broad region categories—association, sensorimotor or subcortical—also varied across the three CLA neuron populations. Such measurements provide us with a general sense of the functional attributes of each CLA network. For example, although retM1 neurons only innervated a small number of sensorimotor regions (Figure 9D, Sensorimotor), the network profile of retM1 neurons was heavily focused on sensorimotor targets (Figure 9E; retM1). This difference indicates that retM1 innervation was concentrated in a small number of sensorimotor targets, implying that retM1 output may serve a more focused sensorimotor function. In contrast, retBLA neurons innervated a large number of subcortical targets despite a low fraction of axonal densities in the subcortical category. This indicates that retBLA neurons may uniquely serve to distribute diffuse outputs to the subcortex.

Among the three broad region categories, all three CLA networks heavily innervated association cortices (>40% axonal density fraction, Figure 9E, green bars) with a similar network span (≥10 regions, Figure 9D, Association). This indicates that innervating association cortices could be a uniform feature across the entire CLA complex.

To quantify the degree of difference and similarity between the different CLA networks, we compared the fraction of axonal density in each brain region between the three projection-defined populations *via* a One-Way ANOVA for each brain region. Innervated regions were then split based on whether they significantly differed (Figure 9F, Different—*p* < 0.05, Similar—*p* ≥ 0.05), and subsequently totaled for each broad region category (Figure 9F). Reflecting the large network differences seen in the earlier broad comparisons, a large number of regions belonging to each category significantly differed (Figure 9F, Different). Both sensorimotor and association categories had more regions that differed (Figure 9F, Different) compared to regions that were similar (Figure 9F, Similar). Conversely, an equal number of subcortical regions were similarly and differently innervated (Figure 9F, Subcortical; Similar = 10, Different = 10). These broad comparisons establish that the three topologically distinct CLA populations—retRSC, retM1, and retBLA—largely differ in their cortical projections but overlap more in their subcortical innervation.

### Functional Segregation of Networks of CLA Neurons Projecting to RSC, M1, and BLA

**Figure 10:**
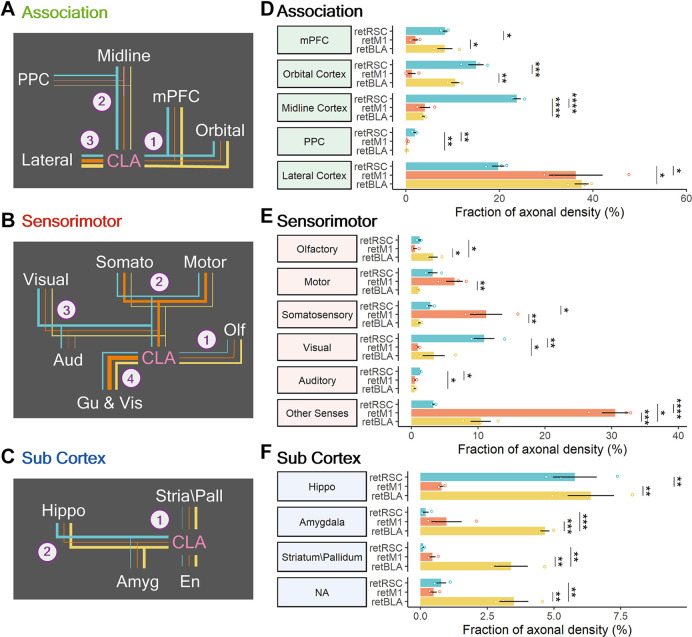
Modularity of CLA neurons projecting to RSC, M1, and BLA. **(A–C)** Summary of connection strengths and trends in each broad region category. Line colors represent the different CLA projection populations: RSC-projecting (retRSC—teal), M1-projecting CLA neurons (retM1—orange), and BLA-projecting (retBLA—yellow). Line thickness represents strength of innervation by the different CLA neuron populations. Numbered branches represent different innervation trends. e.g., Trend A-1: retRSC and retBLA both project heavily to mPFC and Orbital. Trend A-2: Only retRSC heavily projects to Midline and PPC. **(A)** For association regions. **(B)** For sensorimotor regions. **(C)** For the subcortical category. **(D–F)** Axonal densities clustered into functional groups to compare innervation preferences across the three CLA projection populations: association cortices **(D)**, sensorimotor cortices **(E)**, and subcortical category **(F)**. Colors represent different CLA populations: retRSC (teal), retM1 (orange), and retBLA (yellow). Statistical testing was based on *post-hoc* Tukey’s HSD after ANOVA. Significance values: * < 0.05, ** < 0.005, *** < 0.001 and **** < 0.0001.

**Figure 11:**
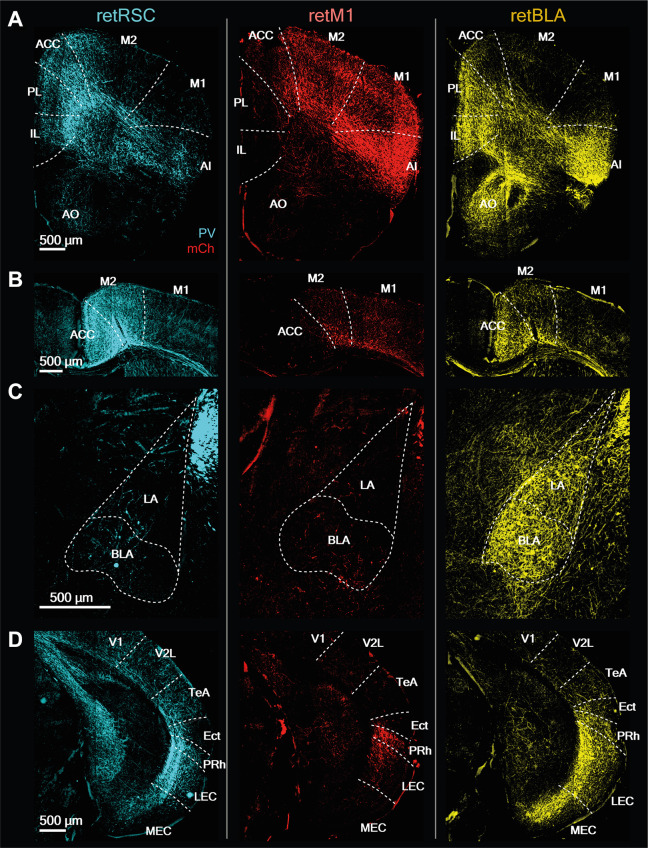
CLA neurons projecting to RSC, BLA, and M1 have distinct innervation patterns. Representative images from prominent innervation targets illustrating the diversity of innervation of RSC-projecting (retRSC), M1-projecting (retM1), and BLA-projecting (retBLA) CLA neuron populations. Each column of images belongs to one of the three CLA projection populations: retRSC, retM1, and retBLA (right to left). **(A)** Anterior section. PL, Prelimbic; IL, Infralimbic; M1, M2, Primary, and secondary motor cortex; AI, Agranular insula; AO, Anterior olfactory nucleus. **(B)** Dorsal-midline structures. ACC, Anterior cingulate cortex. **(C)** Amygdala. BLA, LA, Basolateral, lateral amygdala. **(D)** Posterior section. V1, V2, Primary, secondary visual cortex. TeA, Temporal association cortex; Ect, Ectorhinal cortex; Prh, Perirhinal cortex; LEC, MEC, Lateral, medial entorhinal cortex.

To determine whether the differences in network distribution reflect functional segregation between the CLA networks, we divided each broad region category into functional groups and compared their axonal densities across the three CLA projection populations—ANOVA followed by *post-hoc* Tukey’s HSD. Among the association targets, the regions innervated by either of the three populations can be divided into the medial mPFC (PL, IL), ORB, midline cortex (FrP, ACC and RSC), PPC and lateral cortices (AI, Ect, Prh, TeA, Pir). Preference for association-related functional groups could be divided into three main trends (Figures 10A,D). Firstly, both the retRSC and retBLA neurons showed heavy innervation of the mPFC and ORB compared to the retM1 neuron population (Figures 10A-1, Figure 9D, 11A—PL, IL). Secondly, only the retRSC neuron population heavily innervates the midline cortex and PPC (Figures 10A-2, 9D, 11A,B—ACC). Finally, both retM1 and retBLA neuron populations innervate the lateral cortices more than the retRSC neuron population (Figures 10A-3,D, 11A—AI). These differences indicate that although all three CLA neuron populations project to all association regions, each preferentially innervates specific association targets.

Sensorimotor target regions were divided into the five main sensorimotor modules, while the Gu and Vis were grouped under Other Senses (Figures 10B,E). Each CLA projection population showed clear preferences for different sensorimotor modalities. retBLA neurons preferentially innervated the olfactory cortices (Figures 10B-1,E, 11A—AO), while retM1 neurons preferentially innervated somatic cortices—somatosensory and motor cortices (Figures 10B-2,E, 11A—M2, M1), and retRSC neurons preferentially innervated the visual and auditory cortices (Figures 10B-3,E, 11D—V1, V2L). For the Gu and Vis cortices, innervation from the retM1 population was the highest followed by the retBLA population and the retRSC population (Figures 10B-4,E). These innervation differences reveal clear sensorimotor preferences for each CLA projection-population, indicating that these topologically distinct CLA populations influence non-overlapping sensorimotor modalities.

Lastly, the subcortical targets were divided into four main functional groups: hippocampus-related, amygdala-related, striatum/pallidum, and NA. The NA group includes only the En, because its functional classification is unknown. retBLA neurons innervated all of the subcortical groups significantly more than retM1 and retRSC neurons (Figures 10C-1,F, 11C–BLA), except for the hippocampus-related targets which were also strongly innervated by retRSC neurons (Figures 10C-2,F, 11D—MEC, LEC). These differences indicate that, except for hippocampus-related regions, all other subcortical targets were uniquely innervated by retBLA neurons residing in the vCLA shell.

**Figure 12:**
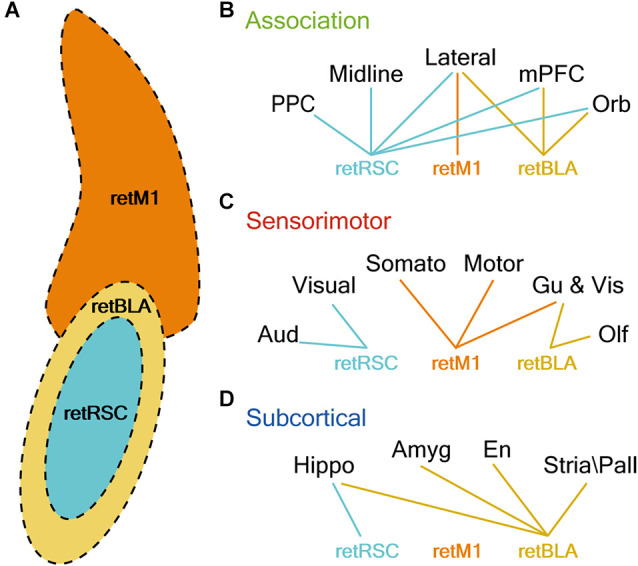
CLA neurons projecting to RSC, M1, and BLA reside in distinct topological locations and strongly innervate distinct sets of brain regions. **(A)** Illustration of CLA subzones. Colors represent the topological location CLA neurons projecting to M1 (retM1, orange), BLA (retBLA, yellow), and RSC (retRSC, teal). **(B–D)** Lines represent connection preferences of different CLA projection neurons. Connection preferences are divided into three functional categories: Association **(A)**, Sensorimotor **(B)**, and Subcortical **(C)**. retRSC neurons strongly innervate all association cortices, but only the visual and auditory cortex (sensorimotor) and hippocampal formation (subcortical). retM1 neurons strongly innervate only the lateral association (association), somatosensory, motor, gustatory and visceral cortices (sensorimotor). retBLA neurons strongly innervate only the frontal and lateral association (association), olfactory, gustatory and visceral cortices (sensorimotor), and all subcortical targets (subcortical).

By comparing the density of innervation of different functional groups by the three CLA projection neuron populations, we established that the topological organization within the CLA complex reflects the functional segregation of output networks (Figure 12). Each of the three CLA populations clearly exhibits minimally overlapping projection preferences, suggesting that the output of the CLA complex can be functionally separated into at least three distinct networks: vCLA core, vCLA shell, and dCLA (Figure 12A). Although networks from each of the 3 CLA populations heavily innervate a specific subset of regions across the brain, collectively the networks cover almost the entire cortex and therefore should be capable of significantly influencing all association and sensorimotor modules.

## Discussion

We have characterized the axonal networks of CLA neurons residing in different topological zones. By systematically labeling three projection-defined populations of CLA neurons and mapping their output networks, we established that CLA neurons projecting to either the RSC, M1, or BLA reside in distinct topological zones within the CLA and project their axons to distinct network arrays. We, therefore, conclude that topologically distinct CLA neurons have output networks that are both broad and functionally distinct, collectively enabling global coordination of brain function.

Our results reconcile two different views of CLA organization that have influenced our conceptualization of CLA function. Reports of multi-region output (Kitanishi and Matsuo, 2017; Zingg et al., 2018) and brain-wide axon arborizations from single CLA neurons (Zingg et al., 2018; Peng et al., 2021) suggest that the CLA may function *via* broadcasting, advocating for a singular role in coordinating brain-wide functions such as consciousness (Crick and Koch, 2005) or multisensory perceptual integration (Smythies et al., 2012). In contrast, reports of topological segregation between CLA neurons that project to different targets (Smith et al., 2019; Marriott et al., 2021) suggest that the CLA is modular, with specialized regions designated to unique computational purposes (Chia et al., 2020; Marriott et al., 2021). Although some of these studies contain hints that both features may co-exist within the CLA (Kitanishi and Matsuo, 2017; Zingg et al., 2018; Marriott et al., 2021), beyond the analysis of RSC-projecting neurons by Zingg et al. (2018); none have demonstrated or characterized the extent of broadcasting in different CLA zones. We have conclusively reconciled these ideas by demonstrating that both concepts are partially correct: the CLA is segregated into distinct topological modules, but each has an ability to broadcast to large downstream networks that minimally overlap. Together, these modules could allow the CLA to effectively influence large brain-wide networks, potentially allowing rapid switching of brain states, or even to coordinate different brain-wide computations in parallel.

### Different Zones for Projection Neurons Within the CLA

A recent comprehensive study of projection-based CLA topology (Marriott et al., 2021) suggests that CLA neurons are organized in a gradient-like fashion, with neurons that project in a rostral-caudal gradient arranged in a dorsal-ventral map. Our results are partially consistent with this mapping scheme: we found that neurons projecting to M1 are localized in a more dorsal region compared to neurons projecting to the RSC (M1 is more anterior than RSC). However, BLA-projecting neurons appear to ignore such mapping rules: BLA-projecting neurons surround the RSC-projecting neurons despite the BLA being more posterior than the RSC. This suggests that subcortical targets may follow a different organizational scheme.

In contrast to previous studies, Marriott et al. (2021) also demonstrated convincingly that CLA neurons projecting to different regions display a gradient-like distribution within the CLA and exhibit overlapping topologies. However, some populations appear to be more distinctly separated compared to others. In particular, previous reports have suggested that CLA neurons projecting to the RSC, M1, and BLA reside in distinct topological zones within the CLA (Jackson et al., 2018; Zingg et al., 2018). Our side-by-side comparisons clearly confirm such organization and emphasize the topological distinction between these three CLA projection neuron populations. RSC-projecting CLA neurons localize within the PV-rich vCLA core, while BLA-projecting CLA neurons localize in the vCLA shell, and M1-projecting neurons form a separate nucleus above the PV-rich vCLA core, the dCLA (Figure 12A). We also show that these topological boundaries are maintained throughout the rostral-caudal axis, scaling together with the claustrum and ending as the hippocampus emerges (Figure 2). This is in contrast to a previous suggestion that the CLA is segregated into different functional domains in the rostral-caudal direction (Goll et al., 2015). Our results indicate that the CLA is organized mainly in the coronal plane, and this topological arrangement is maintained throughout the rostral-caudal axis. The distinct topological localization of these three projection-defined CLA neuron populations made them ideal targets in our effort to discern potential differences in the distribution of their axonal networks.

### CLA Neurons Projecting to RSC, M1, and BLA All Exhibit Extensive Axon Networks

CLA neurons are known to project to multiple targets (Kitanishi and Matsuo, 2017; Wang et al., 2017; Zingg et al., 2018; Marriott et al., 2021; Peng et al., 2021). However, systematic mapping of output targets, based on CLA neuron topology, has only been shown for neurons residing in the vCLA core (Zingg et al., 2018). We independently replicated the results of Zingg et al. (2018) by examining the retRSC population of the vCLA core and extended it by looking at neurons in the dCLA (retM1 neurons) and the vCLA shell (retBLA neurons). Our results provide the first evidence that neurons residing in the dCLA and vCLA shell also project to multiple targets. Although some studies have shown that M1-projecting CLA neurons can project to multiple targets (Kitanishi and Matsuo, 2017; Marriott et al., 2021), none have described these projections in the context of a recent topological framework that segregates the CLA complex into dCLA, vCLA, and dEN (Smith et al., 2018).

We observed that all three projection-defined neuron populations innervated multiple other brain regions in addition to their initial targets. Axonal expression of mCherry in axons beyond our initial sites of retrograde virus injection indicates that the same CLA neurons that innervate the initial target regions also send axon branches to the other labeled regions. In total, the retRSC population innervated 37 regions, while the retBLA population innervated 48 regions and the retM1 population innervated 19 regions. These results establish that all three CLA neuron populations have extensive axon networks, which indicates that broadcasting may be a general feature of CLA information processing.

Our results also support recent reports of cross-hemispheric axon networks from single CLA neurons (Peng et al., 2021). We observed CLA innervation in both ipsilateral and contralateral brain regions, indicating that CLA neurons project to both hemispheres. However, innervation from all three projection-defined CLA neuron populations was largely ipsilateral, with only sparse innervation in a few contralateral targets. The heavy ipsilateral innervation indicates that CLA output is largely restricted to influencing ipsilateral brain activity. Despite this, the presence of contralateral innervations suggests that the CLA may be involved in functions requiring interhemispheric communication, such as coordination of movement or tracking objects across 3D space.

### Divergent Networks of CLA Projection Neurons

Our comparison of the different networks revealed several trends that suggest functional specialization of CLA neurons residing in different topological zones. By comparing innervation densities in different functional regions, we found that neurons in each topological zone within the CLA complex—vCLA core, vCLA shell, and dCLA—not only preferentially innervate different sets of downstream targets but also vary in the extent of innervation (Figures 10, 12).

Among the association targets (Figure 12B), there was a clear dominance of the vCLA core network for innervating association regions, while the vCLA shell and dCLA networks only innervated specific association targets. These trends suggest that vCLA core neurons have a more global, association-related function, in comparison to highly specific association-related functions for the non-core zones. For the sensorimotor targets, we observed a distinct dominance of each network for specific sensorimotor modules (Figure 12C). These sensorimotor preferences may represent intrinsic sensorimotor-function pairings, where each CLA subzone performs functions that employ different sensorimotor modalities. Considering subcortical innervation (Figure 12D), only the vCLA shell network heavily innervated all subcortical targets. Although the vCLA core network also exhibited heavy innervation of hippocampus-related structures, its primary target was the entorhinal cortex, which is both a hippocampus-related structure and an association cortex. Hence, the vCLA shell network may be the primary link between the CLA and subcortical targets.

From the perspective of individual populations, retRSC neurons residing in the vCLA core heavily innervated visual and auditory sensorimotor cortices and all association cortices, including the entorhinal cortex. This unique preference suggests that the vCLA core population may be involved in attention-related and salience-related processing that occurs at fast temporal scales, requiring the coordination of both visual and auditory cortices with association cortices across the board. This network preference observed in vCLA core neurons is in line with recent studies showing deficits in attention and salience-related functions following the perturbation of CLA neurons (Atlan et al., 2018; Terem et al., 2020).

In contrast, the retM1 population residing in the dCLA heavily innervates the motor, somatosensory, gustatory, and visceral sensorimotor cortices, but only heavily innervates the lateral association cortices (mainly the agranular insula). These preferences suggest that the dCLA population may primarily be involved in coordinating somatic-and-visceral-related regions that might be connected to insula function: autonomic control, interoception, pain or somatic processing (Lu et al., 2016; Gogolla, 2017; Uddin et al., 2017). Lastly, the retBLA neuron population residing in the vCLA shell heavily innervated only the frontal association cortices, olfactory cortex, and several limbic-related subcortical regions. These preferences suggest that the vCLA shell population may primarily be involved in coordinating limbic-related functions such as affective processing, where olfaction plays an important role in rodents.

These differences in axonal network preferences provide strong evidence for a modular CLA, where topological organization reflects a functional segregation of different axonal networks/distribution channels. We established that the CLA can be divided into at least three topologically distinct zones that have unique network preferences which possibly allow parallel or competing processing of different global functions. It will be interesting to specifically perturb these CLA subpopulations during behavior and observe potential differences in function: it is likely that each CLA subzone is specialized to implement different behavioral functions that require coordination of multiple brain networks.

### Conclusions

Advances in CLA targeting techniques have enabled a plethora of studies that have begun to uncover the poorly-understood functions of the CLA. However, it has proven difficult to reconcile differences in the outcome of these studies because of the wide range of anatomical and genetic targeting methods that have been used. Each targeting method has interrogated different, but likely overlapping, populations of CLA neurons that reside in multiple CLA subzones. The topology-based functional segregation in the CLA that we have uncovered reveals the difficulty in comparing results that include CLA neurons from different topological subzones. In the future it will be imperative to keep in mind the zonal structure of the CLA, specifically to discern possible differences in the function of different CLA neuron subpopulations within different subzones.

Our experiments have established a fundamental organizational property of claustral topology. Retrograde tracing and axonal mapping of M1-, RSC- and BLA-projecting claustral neurons revealed distinct localization of these neurons into separate claustral subregions and uncovered unique axon projection networks with distinct innervation trends. The distinctive differences in axonal networks suggest that CLA neurons residing in different topological zones are involved in drastically different forms of computation and potentially process and disseminate different types of information. Our discoveries, in conjunction with reports of topologically organized inputs to the CLA (Atlan et al., 2017; Chia et al., 2020), advance understanding of the functional framework within the claustral complex and will motivate future characterization and reporting of claustral subregions. Our discovery of distinct network preferences also will help guide future efforts to deduce the computational function of each CLA neuron subpopulation.

## Data Availability Statement

The raw data supporting the conclusions of this article are freely accessible at the NTU Data Repository https://doi.org/10.21979/N9/FCPGSA.

## Ethics Statement

The animal study was reviewed and approved by the NTU Institutional Animal Care and Use Committee.

## Author Contributions

GH designed and performed the experiments, analyzed and interpreted the data, and wrote the article. GA designed the experiments, interpreted the data, wrote the article, and provided funding. All authors contributed to the article and approved the submitted version.

## Conflict of Interest

The authors declare that the research was conducted in the absence of any commercial or financial relationships that could be construed as a potential conflict of interest.

## Publisher’s Note

All claims expressed in this article are solely those of the authors and do not necessarily represent those of their affiliated organizations, or those of the publisher, the editors and the reviewers. Any product that may be evaluated in this article, or claim that may be made by its manufacturer, is not guaranteed or endorsed by the publisher.

## Footnotes

^1^ https://imagescience.org/meijering/software/featurej/

## Abbreviations

ACC: Anterior cingulate cortex
AI: Agranular insula cortex
amV: anteromedial visual area
aPrS: Area prostriata
BLA: Basolateral amygdala
EC: Entorhinal cortex
Ect: Ectorhinal cortex
En: Endopiriform nucleus
FrP: Frontal Pole
Gu: Gustatory cortex
IL: Infralimbic cortex
LEC: Lateral entorhinal cortex
M1: Primary motor cortex
M2: Secondary motor cortex
MEC: Medial entorhinal cortex
mPFC: medial prefrontal cortex
ORB: Orbital Area
Olf: Olfactory cortex
Pir: Piriform cortex
PL: Prelimbic cortex
pmV: posteromedial visual area
PoRh: Postrhinal area
PoS: Postsubiculum
PPC: Post parietal cortex
PreS: Presubiculum
Prh: Perirhinal cortex
retBLA: BLA-projecting CLA neurons
retM1: M1-projecting CLA neurons
retRSC: RSC-projecting CLA neurons
Sub: Subiculum
S1: Primary somatosensory cortex
TeA: Temporal association cortex
V2: Secondary visual cortex
Vis: Visceral cortex.
